# Supplementary material for: Mutations primarily alter the inclusion of alternatively spliced exons
Source: eLife. 2020 Oct 28;9:e59959. doi: 10.7554/eLife.59959 (PMC7673789; doi:10.7554/eLife.59959)
Supplement: Transparent reporting form [file elife-59959-transrepform.docx]

***eLife’s* transparent reporting form**

We encourage authors to provide detailed information *within their submission* to facilitate the interpretation and replication of experiments. Authors can upload supporting documentation to indicate the use of appropriate reporting guidelines for health-related research (see [EQUATOR Network](http://www.equator-network.org/%20)), life science research (see the [BioSharing Information Resource](https://biosharing.org/)), or the [ARRIVE guidelines](http://www.plosbiology.org/article/info:doi/10.1371/journal.pbio.1000412) for reporting work involving animal research. Where applicable, authors should refer to any relevant reporting standards documents in this form.

If you have any questions, please consult our Journal Policies and/or contact us: [editorial@elifesciences.org](mailto:editorial@elifesciences.org).

**Sample-size estimation**

- You should state whether an appropriate sample size was computed when the study was being designed
- You should state the statistical method of sample size computation and any required assumptions
- If no explicit power analysis was used, you should describe how you decided what sample (replicate) size (number) to use

Please outline where this information can be found within the submission (e.g., sections or figure legends), or explain why this information doesn’t apply to your submission:

In our experiments we performed deep mutagenesis of two different exons, so the sample size for each experiment is the total number of possible single-nucleotide substitutions in these exons.

We chose two different highly-included exons that fall into two different categories of highly included exons: one is the inferred ancestor of a present-day alternative exon, the other is a constitutive exon that is highly included in many different vertebrate species. Although 2 is a small sample size to make generalizable conclusions, we performed genome-wide analyses to validate our results.

**Replicates**

- You should report how often each experiment was performed
- You should include a definition of biological versus technical replication
- The data obtained should be provided and sufficient information should be provided to indicate the number of independent biological and/or technical replicates
- If you encountered any outliers, you should describe how these were handled
- Criteria for exclusion/inclusion of data should be clearly stated
- High-throughput sequence data should be uploaded before submission, with a private link for reviewers provided (these are available from both GEO and ArrayExpress)

Please outline where this information can be found within the submission (e.g., sections or figure legends), or explain why this information doesn’t apply to your submission:

Replicate information about our deep mutational scanning experiments can be found in the methods section.

Information regarding the number of technical replicates in the ancestral *FAS* exon 6 library can be found in the methods section titled “Ancestral *FAS* exon 6 input library”.

Information about the number of biological replicates in the ancestral *FAS* exon 6 library can be found in the methods section titled “Cell transfection and generation of output libraries”.

Information about the number of replicates in the *PSMD14* exon 11 library is found in the methods section titled “PSMD14 exon 11 library”.

**Statistical reporting**

- Statistical analysis methods should be described and justified
- Raw data should be presented in figures whenever informative to do so (typically when N per group is less than 10)
- For each experiment, you should identify the statistical tests used, exact values of N, definitions of center, methods of multiple test correction, and dispersion and precision measures (e.g., mean, median, SD, SEM, confidence intervals; and, for the major substantive results, a measure of effect size (e.g., Pearson's r, Cohen's d)
- Report exact p-values wherever possible alongside the summary statistics and 95% confidence intervals. These should be reported for all key questions and not only when the p-value is less than 0.05.

Please outline where this information can be found within the submission (e.g., sections or figure legends), or explain why this information doesn’t apply to your submission:

Summary statistics (median effect size of mutations in ΔPSI units, and the proportion of mutations with an effect greater than 10 PSI units) are shown throughout the results section (subsections “Deep mutagenesis confirms mutations rarely have large effects in exons with high inclusion levels”, “Exonic mutations primarily alter the inclusion of exons with intermediate levels of inclusion”, “Common variants also primarily alter the inclusion of exons with intermediate levels of inclusion”, “Estimating the distribution of exonic mutational effects for the human genome”, “Intronic mutations also rarely alter the inclusion of highly-included exons”, “Splice site mutations do frequently alter the inclusion of highly-included exons”).

Some t tests were performed in the results section titled “Mutations have stronger effects in highly-included alternatively spliced exons than in constitutive exons” and the raw p values are shown unless they fall below R’s lower limit of 2.2e–16.

Data about genome-wide mutations and variants was split into groups and visualised using violin plots in figures 3, 4, 5, 6 and 8. The number of mutations in each group is shown on the upper right hand of each violin plot. In addition, when the number of data points was below 10 (figure 8C), the raw data is also shown.

(For large datasets, or papers with a very large number of statistical tests, you may upload a single table file with tests, Ns, etc., with reference to sections in the manuscript.)

**Group allocation**

- Indicate how samples were allocated into experimental groups (in the case of clinical studies, please specify allocation to treatment method); if randomization was used, please also state if restricted randomization was applied
- Indicate if masking was used during group allocation, data collection and/or data analysis

Please outline where this information can be found within the submission (e.g., sections or figure legends), or explain why this information doesn’t apply to your submission:

In our paper, group allocation occurs in the results section titled “Mutations have stronger effects in highly-included alternatively spliced exons than in constitutive exons”, in which all exons in the genome are split into “constitutive” and “alternative” exons. This classification is explained in the first paragraph of this section.

**Additional data files (“source data”)**

- We encourage you to upload relevant additional data files, such as numerical data that are represented as a graph in a figure, or as a summary table
- Where provided, these should be in the most useful format, and they can be uploaded as “Source data” files linked to a main figure or table
- Include model definition files including the full list of parameters used
- Include code used for data analysis (e.g., R, MatLab)
- Avoid stating that data files are “available upon request”

Please indicate the figures or tables for which source data files have been provided:

Sequencing data produced in this experiment has been uploaded to GEO (number GSE151942) as described in the “Data Access” section of this manuscript.

Code used for data analysis has been uploaded to Github (https://github.com/lehner-lab/Constitutive_Exons).
